# Evaluation of Oropharyngeal Dysphagia in Older Patients for Risk Stratification of Pneumonia

**DOI:** 10.3389/fimmu.2021.800029

**Published:** 2022-02-02

**Authors:** Tai-Han Lin, Chih-Wei Yang, Wei-Kuo Chang

**Affiliations:** ^1^ Department of Pathology and Graduate Institute of Pathology and Parasitology, Tri-Service General Hospital, National Defense Medical Center, Taipei, Taiwan; ^2^ Division of Gastroenterology, Department of Internal Medicine, Tri-Service General Hospital, National Defense Medical Center, Taipei, Taiwan

**Keywords:** older patients, oropharyngeal dysphagia, aspiration pneumonia, percutaneous endoscopic gastrostomy, nasogastric tube, tube feeding, enteral feeding

## Abstract

**Objective:**

Nasogastric tube (NGT) and percutaneous endoscopic gastrostomy (PEG) are widely used techniques to feed older patients with oropharyngeal dysphagia. Aspiration pneumonia is the most common cause of death in these patients. This study aimed to evaluate the role of oropharyngeal dysphagia in older patients on long-term enteral feeding for risk stratification of pneumonia requiring hospitalization.

**Methods:**

We performed modified flexible endoscopic evaluation of swallowing to evaluate oropharyngeal dysphagia in older patients and conducted prospective follow-up for pneumonia requiring hospitalization. A total of 664 oral-feeding patients and 155 tube-feeding patients were enrolled. Multivariate Cox analysis was performed to identify risk factors of pneumonia requiring hospitalization.

**Results:**

Multivariate analyses showed that the risk of pneumonia requiring hospitalization significantly increased in older patients and in patients with neurological disorders, tube feeding, and oropharyngeal dysphagia. Subgroup analysis revealed that the risk of pneumonia requiring hospitalization was significantly lower in patients with PEG than in those with NGT among the patients with oropharyngeal dysphagia (adjusted hazard ratio 0.26, 95% confidence interval: 0.11–0.63, *P* = 0.003).

**Conclusions:**

For older patients with oropharyngeal dysphagia requiring long-term enteral tube feeding, PEG is a better choice than NGT. Further research is needed to elucidate the role of oropharyngeal dysphagia in enteral feeding in older patients.

## Introduction

Population aging is a global issue, and it is estimated that the older population will reach approximately 1.5 billion in 2050 (1). Degeneration and multiple comorbidities accompany as people age, and approximately 13%–81% of older patients are affected by oropharyngeal dysphagia (1–4). This condition is common particularly in patients with neurological disorders (3, 4). Patients with oropharyngeal dysphagia may require enteral tube feeding if they cannot meet their nutritional needs orally (5). Such patients may face greater socioeconomic issues as the prevalence rates of stroke, dementia, and esophageal motility disorder increase with older age (4, 6, 7).

The two commonly used methods of enteral tube feeding are nasogastric tube (NGT) and percutaneous endoscopic gastrostomy (PEG) (8). Aspiration pneumonia is the most common cause of death in these patients with enteral tube feeding (9, 10), with estimated incidence rates of 12%–87% for NGT (11–15) and 9%–52% for PEG (12–15). At present, NGT is recommended for temporary enteral nutrition lasting less than 4 weeks, whereas PEG is recommended for cases longer than 4 weeks (16). However, in Asian counties, NGT is more frequently selected than PEG for long-term enteral feeding. Possible explanations for this choice include refusal of interventional surgical procedures by the patient’s family due to considering patients has already been tortured by long-term morbidity or disability, desire to follow the custom of maintaining the “whole corpse” for burial purposes, and concerns related to complications after the PEG procedure (17–19).

The risks of aspiration pneumonia in older patients rise significantly with oropharyngeal dysphagia (20, 21). Meta-analyses have not conclusively determined whether oropharyngeal dysphagia is a risk for aspiration pneumonia in older patients on enteral feeding and whether PEG is better than NGT (15, 22). However, this may be due to the high level of statistical heterogeneity among analyzed studies. Therefore, oropharyngeal dysphagia should be evaluated and subgroup analysis regarding the risk of pneumonia in older patients on long-term enteral feeding, especially in regions in favor or NGT over PEG, should be performed.

Flexible endoscopic evaluation of swallowing (FEES) is a well-documented standard method for evaluating oropharyngeal dysphagia (23, 24). This method allows assessment of aspiration risk by direct visualization of test material accumulating in the pharyngolaryngeal region or entering the vocal cords (23–26); however, adverse events such as epistaxis, vasovagal response, and laryngospasm can develop during the procedure (27). Furthermore, this method can only be performed if the patient has adequate physical and cognitive function to cooperate with degulgitation, and thus cannot be properly applied in patients with advanced neurological diseases such as dementia, Parkinson’s disease, or stroke (24–26). In contrast, a modified version of FEES that involves additional pharyngolaryngeal examination during upper gastrointestinal (UGI) endoscopy is easy and bearable to patients, and may thus help in stratifying the severity of oropharyngeal dysphagia according to the pooling of secretions in the pharyngolaryngeal region (24).

Aspiration pneumonia could develop if aspiration of potential pathogenic gastric contents or oropharyngeal secretions into the larynx or respiratory tract, which is common in older adults with dysphagia (28). As more residues in the pharyngolaryngeal region were reported in those with NGT due to NGT passes through the upper esophageal sphincter (29–32), we hypothesized that oropharyngeal dysphagia significantly increased the risk of pneumonia requiring hospitalization in older patients with NGT, and that PEG is recommended in preference to NGT for patients with oropharyngeal dysphagia requiring long-term tube feeding.

Previous studies primarily focused on the utility of FEES in evaluating the risk of aspiration pneumonia in patients with oral feeding, whereas studies examining the utility of FEES in evaluating the risks in NGT- and PEG-feeding patients remain limited. This study aimed to evaluate the role of oropharyngeal dysphagia for risk stratification of pneumonia requiring hospitalization in older patients on long-term enteral feeding.

## Materials and Methods

### Patients

We performed modified FEES in older patients who received enteral feeding between January 2015 and July 2020. Additionally, we conducted a prospective follow-up of those requiring hospital admission due to pneumonia. The study was approved by the institutional review board of the Tri-Service General Hospital, No: 2-108-05-136. The participants provided written informed consent.

### Study Design

**Figure 1:**
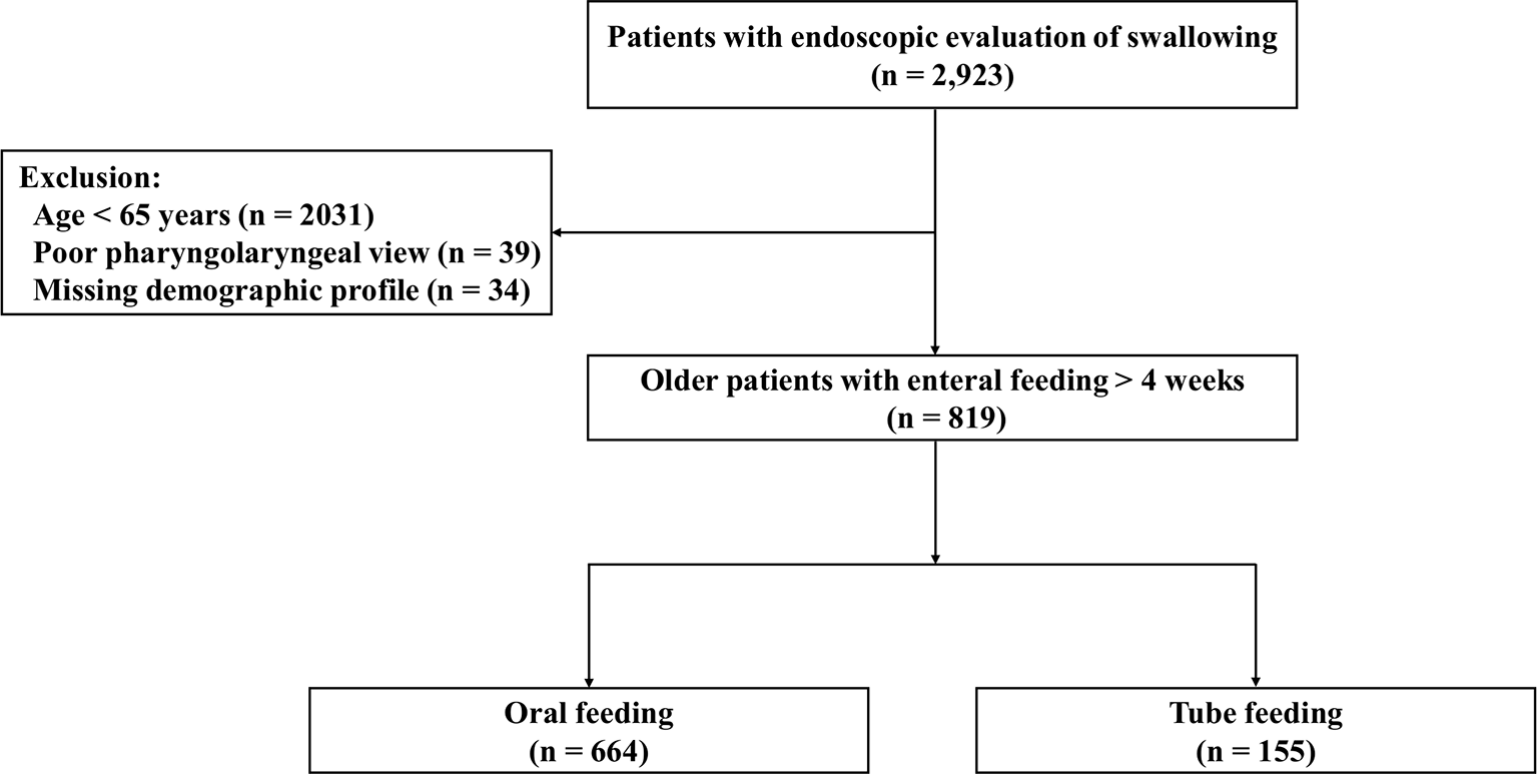
Patient selection flow chart.

Of the 2,923 patients who underwent modified FEES to evaluate oropharyngeal dysphagia (**Figure 1**), 2,031 were excluded because they were below 65 years of age, 39 were excluded because modified FEES provided a poor pharyngolaryngeal view, and 34 were excluded for missing demographic profile data. Of the remaining 819 older patients who required enteral feeding for more than 4 weeks, 664 were enrolled in the oral-feeding group and 155 in the tube-feeding group.

### Demographic and Clinical Data

We recorded the demographic and clinical profiles, such as age, gender, body mass index, reasons for enteral feeding, and pneumonia requiring admission. Reasons for enteral feeding were categorized into two groups according to whether the patient had any neurological disorder, such as Alzheimer’s disease, dementia, Parkinson’s disease, or stroke.

### Modified Flexible Endoscopic Evaluation of Swallowing

**Figure 2:**
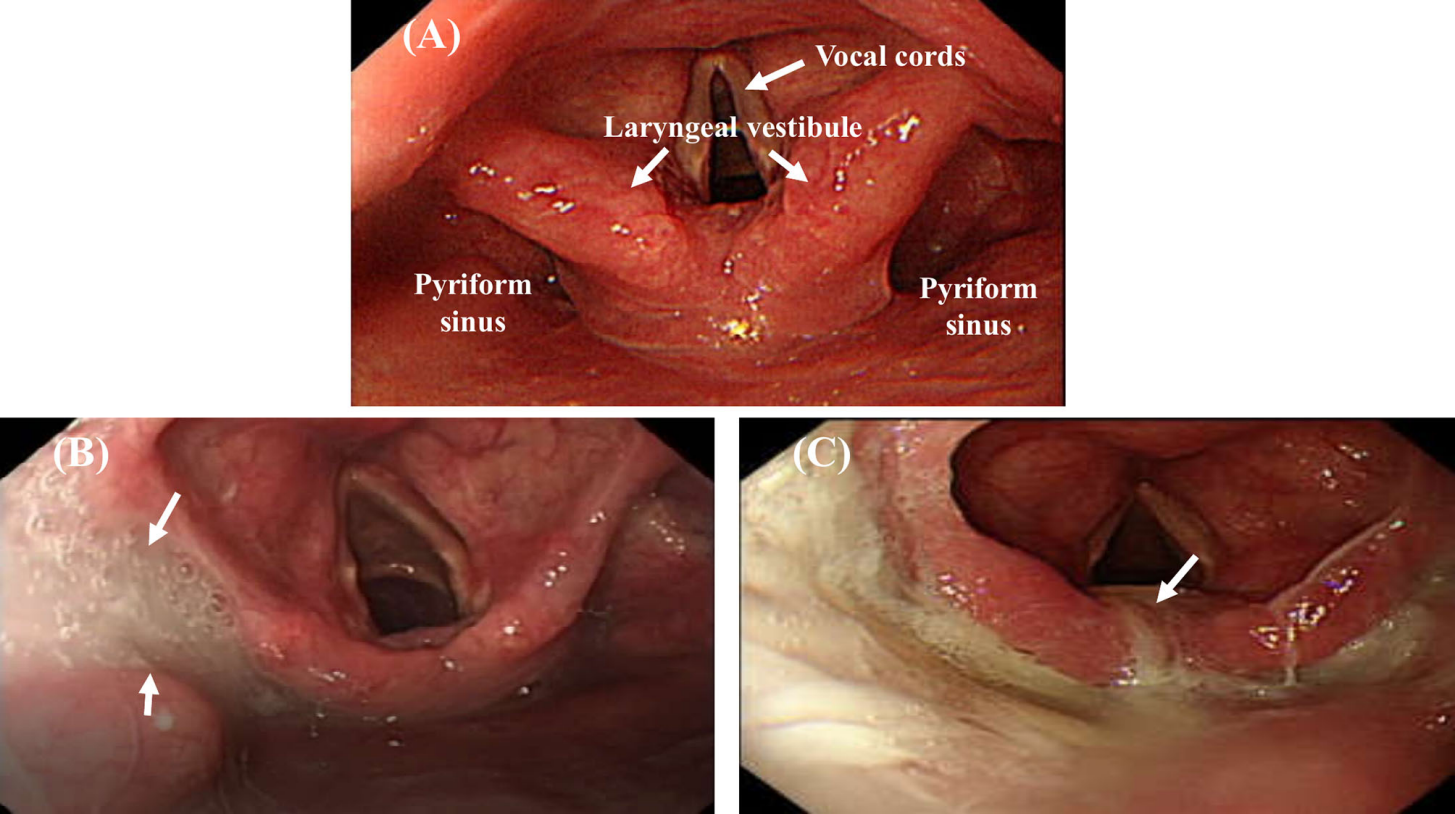
Endoscopic views of pooled secretions (arrow) in the pharyngolaryngeal region. Absence of secretions filling the pharyngolaryngeal region **(A)**. A large amount of pooled secretions in the right side of the pyriform sinus but not entering the laryngeal vestibule **(B)**, and pooled secretions leaking into the laryngeal vestibule **(C)**.

Patients fasted for at least 4 hours before UGI endoscopic examination and were placed in the left lateral decubitus position (24, 33). The endoscope tip was inserted through a mouthpiece with its axis aligned with that of the patient’s esophagus. The endoscope was advanced along the palate midline, rotated slightly, and gently advanced past the uvula with anterior flexion to visualize the pyriform sinus, laryngeal vestibule, vocal cords, and upper part of the trachea (**Figure 2A**). The examination result was recorded using a digital video recorder (HVO-550MD; Sony, Tokyo, Japan) for later analysis and was reviewed frame by frame in slow motion by two endoscopists (WKC and CWY).

### Evaluation of Oropharyngeal Dysphagia

When patients are placed in the left lateral decubitus position during examination, the accumulated secretions may fill the lowest area of the right side of the pyriform sinus, leak into the left side of the pyriform sinus, or flow into the laryngeal vestibule or vocal cords (24). Swallowing frequency is estimated to be about 600 times per day under normal physiological conditions (34). In most patients, no pooling or minimal pooling of secretions in the pharyngolaryngeal region was observed under endoscopy; occasionally, thin, watery secretions or clear bubbles were observed in the pharyngolaryngeal region (24, 25). Oropharyngeal dysphagia was evaluated according to the amount and location of accumulated secretions observed endoscopically in the pharyngolaryngeal region (**Figure 2**) (24, 35). The pooling of secretions in the pyriform sinus was quantified as follows: minimal, < 25%; moderate, 25%–50%; and large, > 50% secretions filling the pyriform sinuses. Endoscopic evidence of oropharyngeal dysphagia was divided into two categories: (1) absence of oropharyngeal dysphagia (< 25% pooled secretions filling the pyriform sinus, **Figure 2A**) and (2) presence of oropharyngeal dysphagia (> 25% pooled secretions filling the pyriform sinus, **Figure 2B**; or pooled secretions entering the laryngeal vestibule, **Figure 2C**) (24, 35).

### Diagnosis of Pneumonia

Pneumonia was diagnosed based on radiological evidence of pulmonary consolidation, shortness of breath, body temperature above 38°C, serum white blood cell count > 10,000/mm^3^, and the requirement for hospitalization within 2 years after the patient was enrolled (36).

### Statistical Analysis

Statistical analyses were performed using SPSS Statistics 22.0 (IBM Inc., Armonk, NY, USA) with a two-sided significance level of 5%. Parametric continuous data were compared using the independent t-test. Categorical data were compared using the Chi-square test or Fisher’s exact test. A univariate and multivariate Cox regression model was applied to determine the association between the clinical-demographic profile and developing pneumonia. The hazard ratio (HR) for pneumonia development was estimated *via* multivariate analysis using the Cox proportional hazards model after adjusting the statistically significant variables in the univariate analysis as potential confounding factors. Survival curves were plotted with the Kaplan–Meier method.

## Results

### Patient Characteristics

A total of 819 older patients were enrolled in this study, with 664 in the oral-feeding group and 155 in the tube-feeding group. Patient characteristics are shown in **Table 1**. The mean age was 76.4 ± 7.5 years in the oral-feeding group and 78.5 ± 8.9 years in the tube-feeding group. Significant differences in age, sex, body mass index, reasons for enteral feeding, and oropharyngeal dysphagia were observed between the two groups.

**Table 1:** Patients characteristics.

Variable	Oral feeding (n = 664)	Tube feeding (n = 155)	*P *Value
Age (years)	76.4 ± 7.5	78.5 ± 8.9	0.006
Sex			0.001
Female	349 (52.6%)	58 (37.4%)	
Male	315 (47.4%)	97 (62.6%)	
Body mass index (kg/m^2^)	23.6 ± 3.8	21.9 ± 3.6	<0.001
Reasons for enteral feeding, no. (%)			<0.001
Neurological disorders	135 (20.3%)	118 (76.1%)	
Non-neurological disorders	529 (79.7%)	37 (23.9%)	
Oropharyngeal dysphagia, no. (%)			<0.001
Absence	613 (92.3%)	96 (61.9%)	
Presence	51 (7.7%)	59 (38.1%)	

### Risk Factors for Pneumonia Requiring Hospitalization

Multivariate analyses showed that the risk of pneumonia requiring hospitalization was significantly increased in patients with older ages (with each year of age increase, adjusted HR 1.03, 95% CI 1.01–1.06, *p* = 0.004), neurological disorders (adjusted HR 2.39, 95% CI: 1.49–3.84, *P <* 0.001), tube feeding (adjusted HR 2.57, 95% CI: 1.61–4.12, *P <* 0.001), and oropharyngeal dysphagia (adjusted HR 1.59, 95% CI: 1.02–2.47, *P* = 0.041) (**Table 2**).

**Table 2:** Multivariable analysis of the factors associated with pneumonia.

Variable	With pneumonia (n = 113)	Without pneumonia (n = 706)	Crude HR (95% CI)	*P* value	Adjusted HR (95% CI)	*P* value
Age (years)	79.8 ± 8.7	76.3 ± 7.6	1.06 (1.03–1.08)	<0.001	1.03 (1.01–1.06)	0.004
Sex, no. (%)						
Female	45 (39.8%)	362 (51.3%)	Reference		Reference	
Male	68 (60.2%)	344 (48.7%)	1.57 (1.08–2.29)	0.018	1.22 (0.83–1.79)	0.302
Body mass index (kg/m^2^)	22.5 ± 3.9	23.4 ± 3.8	0.95 (0.90–0.99)	0.03	0.99 (0.94–1.05)	0.842
Reasons for enteral feeding, no. (%)						
Non-neurological disorders	38 (33.6%)	528 (74.8%)	Reference		Reference	
Neurological disorders	75 (66.4%)	178 (25.2%)	4.92 (3.33–7.27)	<0.001	2.39 (1.49–3.84)	<0.001
Enteral feeding, no. (%)						
Oral feeding	53 (46.9%)	611 (86.5%)	Reference		Reference	
Tube feeding	60 (53.1%)	95 (13.5%)	5.53 (3.82–8.01)	<0.001	2.57 (1.61–4.12)	<0.001
Oropharyngeal dysphagia, no. (%)						
Absence	77 (68.1%)	632 (89.5%)	Reference		Reference	
Presence	36 (31.9%)	74 (10.5%)	3.61 (2.43–5.37)	<0.001	1.59 (1.02–2.47)	0.041

HR, hazard ratio; CI, confidence interval.

### Subgroup Analysis: Presence Versus Absence of Oropharyngeal Dysphagia

**Figure 3:**
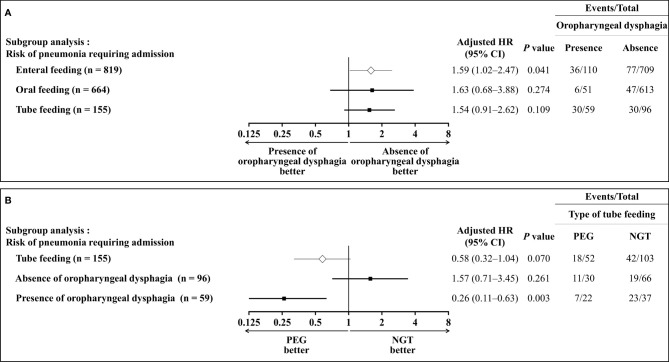
Subgroup analysis of risk of pneumonia requiring admission in older patients with enteral feeding. **(A)** Based on whether the patients had oropharyngeal dysphagia, the risk of pneumonia requiring hospitalization increased significantly in overall enteral feeding patients, but not in oral-feeding and tube-feeding patients. **(B)** Based on the type of tube feeding, PEG did not decrease the risk of pneumonia requiring hospitalization in the overall tube-feeding patients and patients without oropharyngeal dysphagia, but the risk was reduced significantly in patients with oropharyngeal dysphagia.

Subgroup analysis was conducted for patients on enteral feeding based on whether the patients had oropharyngeal dysphagia (**Figure 3A**). Presence of oropharyngeal dysphagia significantly increased the risk of pneumonia in patients with enteral feeding (adjusted HR 1.59, 95% CI: 1.02–2.47, *P* = 0.041). However, it did not increase the risk in patients with oral feeding (adjusted HR 1.63, 95% CI: 0.68–3.88, *P* = 0.274) and in patients with tube feeding (adjusted HR 1.54, 95% CI: 0.91–2.62, *P* = 0.109).

### Subgroup Analysis: PEG Versus NGT

The risk of pneumonia showed no significant difference between PEG and NGT in all tube-feeding patients (adjusted HR 0.58, 95% CI: 0.32–1.04, *P* = 0.070). We conducted subgroup analysis for all enteral feeding patients with tube feeding based on the presence of NGT or PEG long-term feeding (**Figure 3B**). The risk of pneumonia was not significantly different between PEG and NGT (adjusted HR 1.57, 95% CI: 0.71–3.45, *P* = 0.261) in patients with absence of oropharyngeal dysphagia; however, the risk of pneumonia was significantly lower in PEG than NGT in patients with presence of oropharyngeal dysphagia (adjusted HR 0.26, 95% CI: 0.11–0.63, *P* = 0.003).

### Cumulative Proportion of Pneumonia Requiring Admission

**Figure 4:**
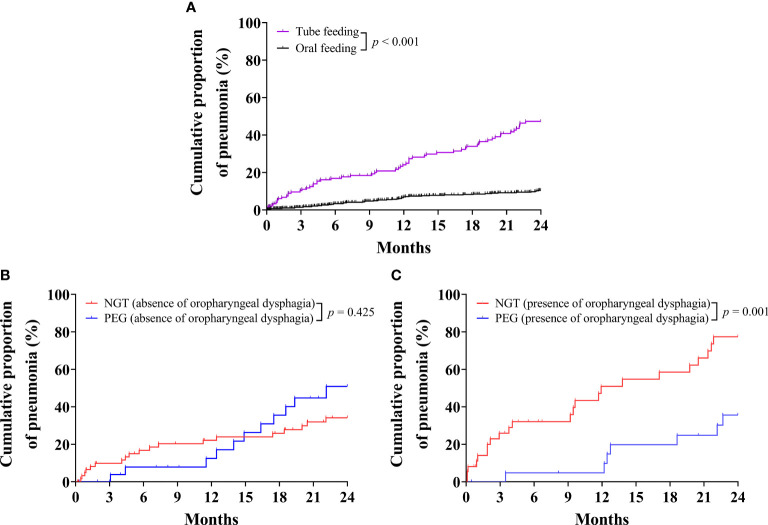
Kaplan–Meier curves for the cumulative proportion of pneumonia requiring hospital admission in older patients. **(A)** Cumulative proportion of pneumonia in older patients for tube-feeding and oral-feeding group. **(B)** Cumulative proportion of pneumonia in older patients without oropharyngeal dysphagia for nasogastric tube (NGT) and percutaneous endoscopic gastrostomy (PEG) groups. **(C)** Cumulative proportion of pneumonia in older patients with oropharyngeal dysphagia for NGT and PEG groups.

Kaplan–Meier analysis indicated that the cumulative proportion of pneumonia was significantly increased in patients with tube feeding compared with oral feeding (*P <* 0.001) (**Figure 4A**). When subgroup analysis patients had absence of oropharyngeal dysphagia, the cumulative proportion of pneumonia was not significantly different between PEG and NGT (*P* = 0.425) (**Figure 4B**). However, when subgroup analysis patients had presence of oropharyngeal dysphagia, the cumulative proportion of pneumonia was significantly decreased in patients with PEG compared with NGT (*P* = 0.001) (**Figure 4C**).

## Discussion

This was a prospective study of older patients requiring long-term enteral tube feeding in a tertiary care center. We found that (1) multivariate analyses showed that older ages, neurological disorders, tube feeding, and oropharyngeal dysphagia were associated with an increased risk of pneumonia requiring hospitalization; (2) oropharyngeal dysphagia significantly increased the risk of pneumonia in patients on long-term enteral feeding; (3) pneumonia significantly decreased in patients with PEG compared to those with NGT among the patients with oropharyngeal dysphagia. The implication of this study is that the evaluation of oropharyngeal dysphagia is crucial for older patients on long-term enteral feeding. In older patients with oropharyngeal dysphagia, PEG is a better choice than NGT for long-term enteral tube feeding.

### Evaluation of Oropharyngeal Dysphagia

Detailed history-taking, physical examination, and FEES are important for evaluating oropharyngeal dysphagia (37), and evaluation of oropharyngeal dysphagia is recommended in patients requiring long-term enteral feeding (33, 38, 39). Digital video recording systems can aid in overcoming the limitations of conventional UGI endoscopy for pharyngolaryngeal examination due to limited photographic recording within a short observation period (24, 40). Furthermore, the recorded video can facilitate the evaluation of oropharyngeal dysphagia by recording protective cough reflex, vocal cord adduction, and movements for clearing secretions during the process (24, 40). Although a swallowing test was not conducted as a part of the traditional methodology of FESS due to the patient characteristics (23–26), we were able to stratify the groups with higher pneumonia risk using modified FESS with UGI endoscopy through the direct visualization of the pharyngolaryngeal region.

### Older Patients With Oropharyngeal Dysphagia

Deterioration in neural control and structural alteration of the pharyngeal region can lead to swallowing difficulties (41), which can gradually worsen with increasing age of people (3, 4, 6, 7). In the current study, the multivariate analyses revealed that the risk of pneumonia requiring hospitalization was significantly higher in older patients (with each year of age increase, adjusted HR 1.03, 95% CI 1.01–1.06, *P* = 0.004) and in patients with neurological disorders (adjusted HR 2.39, 95% CI: 1.49–3.84, *P* < 0.001). Oropharyngeal dysphagia is associated with increased risk of aspiration pneumonia or death (42, 43). The risk of pneumonia requiring hospitalization also increased significantly in patients with oropharyngeal dysphagia (adjusted HR 1.59, 95% CI: 1.02–2.47, *P* = 0.041) in the multivariate analyses in the current study. If oropharyngeal dysphagia is suspected in older patients, referral to a physician or speech therapist is warranted (44). A multidisciplinary team should develop a management plan to monitor, assess, and prevent aspiration pneumonia in patients receiving long-term enteral feeding who are at risk of oropharyngeal aspiration (5, 45). The development of malnutrition might be related to reduced swallowing function due to oropharyngeal dysphagia (20, 46), and further deterioration of laryngeal muscle might lead to a vicious cycle (20, 46). Effective swallowing function and salivary flow are crucial for the clearance of most oropharyngeal pathogens in healthy individuals (47), whereas reduced mechanical clearance might be associated with oropharyngeal residue or bacterial colonization (46, 47). Aspiration pneumonia could develop due to pulmonary aspiration induced by impaired swallowing function, particularly if the aspirated material contains an abundant bacterial load or in the presence of impaired mechanical or immune defense mechanism (48).

### Tube Feeding in Older Patients With Oropharyngeal Dysphagia

Tube feeding is associated with a higher risk of aspiration pneumonia compared to oral feeding (32, 49), and our study showed similar results (adjusted HR 2.57, 95% CI: 1.61–4.12, *P <* 0.001) under multivariate analysis. Although PEG has not shown more favorable outcomes compared to NGT in meta-analyses (15, 22), subgroup analysis in this study demonstrates that the risk of pneumonia was significantly reduced in PEG compared to NGT in older patients with oropharyngeal dysphagia (adjusted HR 0.26, 95% CI: 0.11–0.63, *P* = 0.003), but not in patients without oropharyngeal dysphagia (adjusted HR 1.57, 95% CI: 0.71–3.45, *P* = 0.261). Bacterial colonization and gastroesophageal reflux might increase due to interference with protective cough reflexes in patients with NGT (29–32). The NGT passes through the upper esophageal sphincter, and studies have reported more residues in the pharyngolaryngeal region (29–32), and aspiration of pathogenic bacterial colonization from oropharyngeal secretions could lead to aspiration pneumonia (48). Therefore, in older patients requiring long-term enteral tube feeding with oropharyngeal dysphagia, PEG is a better choice than NGT.

### Management of Oropharyngeal Dysphagia in Older Patients

The management of oropharyngeal dysphagia should include familial support and a multidisciplinary team (45). Strategies for reducing the rate of aspiration pneumonia in patients with oropharyngeal dysphagia include (1) maintaining a semi-recumbent position and turning the patient’s head to one side with the chin down during feeding to reduce the risk of gastric aspiration (50); (2) maintenance of oral hygiene by proper tooth brushing, mechanical oral cleaning, and oral rinsing with chlorhexidine (50–52); (3) dental examination to remove debris, plaque or treat tooth decay (50–52); (4) suctioning of subglottic and oropharyngeal secretions (33, 53); (5) avoidance of oversedation, antipsychotics, and cough suppressants that weaken the cough reflex (50); (6) regular swallowing rehabilitation (54); (7) feeding with PEG rather than NGT in patients requiring long-term tube feeding (24, 33).

### Limitations

Several limitations should be considered when interpreting our results. First, the study was a prospective single-center non-randomized design, and due to the limited sample size, the statistical power is limited. Second, although direct visualization of the pharyngolaryngeal region was feasible with FEES, we could not assess the contents or amounts aspirated during follow-up and determine their relationship to the development of pneumonia. In addition, swallowing trial was not conducted since a significant proportion of enrolled patients had advanced neurological diseases who could not follow instructions during the examination and penetration-aspiration scale was not assessed (55–57). Third, information on body weight variation, feeding intolerance, and gastric residual volume was not available; thus, we could not adjust for these possible confounders (58). However, to the best of our knowledge, the present study is the first prospective study designed to evaluate the optimal choice of long-term tube feeding in older patients with oropharyngeal dysphagia.

## Conclusions

Despite the limitations, this study shows that oropharyngeal dysphagia significantly increased the risk of pneumonia requiring hospitalization in older patients with NGT. PEG is recommended in preference to NGT for patients with oropharyngeal dysphagia requiring long-term tube feeding. Further study will be needed to elucidate the role of oropharyngeal dysphagia in enteral feeding in older patients.

## Data Availability Statement

The datasets presented in this article are not readily available because legal restrictions imposed by the government of Taiwan in relation to the “Personal Information Protection Act”. Requests to access the datasets should be directed to Tri-Service General Hospital.

## Ethics Statement

The studies involving human participants were reviewed and approved by institutional review board of the Tri-Service General Hospital. The patients/participants provided their written informed consent to participate in this study.

## Author Contributions

T-HL and W-KC contributed to conception and design of the study. T-HL organized the database, performed statistical analysis, and wrote the first draft of the manuscript. C-WY and W-KC wrote sections of the manuscript. All authors contributed to manuscript revision, reading, and approval of the submitted version.

## Funding

We are grateful for the financial support provided by the Ministry of National Defense-Medical Affairs Bureau, Tri-Service General Hospital (TSGH-C108-070 and TSGH-D-111080), Taiwan for this study.

## Conflict of Interest

The authors declare that the research was conducted in the absence of any commercial or financial relationships that could be construed as a potential conflict of interest.

## Publisher’s Note

All claims expressed in this article are solely those of the authors and do not necessarily represent those of their affiliated organizations, or those of the publisher, the editors and the reviewers. Any product that may be evaluated in this article, or claim that may be made by its manufacturer, is not guaranteed or endorsed by the publisher.
